# In search of effective therapies to overcome resistance to Temozolomide in brain tumours

**DOI:** 10.20517/cdr.2019.64

**Published:** 2019-12-19

**Authors:** Kaouthar Bouzinab, Helen Summers, Jihong Zhang, Malcolm F. G. Stevens, Christopher J. Moody, Lyudmila Turyanska, Neil R. Thomas, Pavel Gershkovich, Marianne B. Ashford, Emily Vitterso, Lisa C. D. Storer, Richard Grundy, Tracey D. Bradshaw

**Affiliations:** ^1^School of Pharmacy, University of Nottingham, Nottingham NG7 2RD, UK.; ^2^School of Chemistry, University of Nottingham, Nottingham NG7 2RD, UK.; ^3^School of Physics and Astronomy, University of Nottingham, Nottingham NG7 2RD, UK.; ^4^Advanced Drug Delivery, Pharmaceutical Sciences, R&D, AstraZeneca, Macclesfield SK10 4TF, UK.; ^5^School of Medicine, Queen’s Medical Centre, University of Nottingham, Nottingham NG7 2UH, UK.

**Keywords:** Brain cancer, temozolomide-resistance, analogues, drug delivery, apoferritin

## Abstract

Glioblastoma multiforme is the most common and lethal brain tumour-type. The current standard of care includes Temozolomide (TMZ) chemotherapy. However, inherent and acquired resistance to TMZ thwart successful treatment. The direct repair protein methylguanine DNA methyltransferase (MGMT) removes the cytotoxic *O6*-methylguanine (*O*6-MeG) lesion delivered by TMZ and so its expression by tumours confers TMZ-resistance. DNA mismatch repair (MMR) is essential to process *O*6-MeG adducts and MMR-deficiency leads to tolerance of lesions, resistance to TMZ and further DNA mutations. In this article, two strategies to overcome TMZ resistance are discussed: (1) synthesis of imidazotetrazine analogues - designed to retain activity in the presence of MGMT or loss of MMR; (2) preparation of imidazotetrazine-nanoparticles to deliver TMZ preferably to the brain and tumour site. Our promising results encourage belief in a future where better prognoses exist for patients diagnosed with this devastating disease.

## Introduction

**Figure 1:**
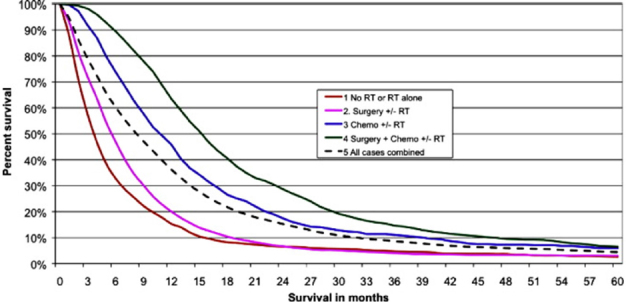
A Kaplan-Meier plot demonstrating survival (in months) of glioblastoma multiforme patients (aged 20-70 years) post diagnosis (2007-2010). The standard of care therapy includes surgery, radiotherapy and Temozolomide chemotherapy treatment. There were significant differences between all treatment groups (*P* < 0.00001). With thanks to Brodbelt *et al.*^[7]^

According to Cancer Research UK statistics from all forms of brain cancers, 10 year survival is 13%; 5 and 1 year survival figures are 19% and 40% respectively. Brain cancers comprise multiple phenotypes and the above statistics, stark as they are, mask appalling and heart-breaking prognoses for example in paediatric diffuse intrinsic pontine glioma (DIPG) and adult glioblastoma multiforme (GBM). A child diagnosed with DIPG today faces the same prognosis as a child diagnosed with this disease 40 years ago. Few children (< 1%) survive beyond 5 years and median survival from diagnosis is 9 months. Tragically, there is currently no effective treatment and no chance of long-term survival. GBM is the most prevalent and aggressive adult central nervous system (CNS) tumour. A high grade (IV) astrocytoma, it is heterogeneous, highly angiogenic, migratory and invasive^[1-6]^. Untreated, median survival from diagnosis of GBM is 4 months. The current standard of care, comprising surgical resection, radiotherapy and temozolomide (TMZ) chemotherapy confers a median survival of ~16 months [Figure 1]^[7]^.

In order to improve progression-free and overall survival, as well as quality of patient life, we need to understand comprehensively the multiple factors that contribute to the pitiful prognoses. In GBM, inherent or acquired tumour resistance thwarts successful treatment; thus knowledge of GBM pathogenesis and biology, mechanisms of action and resistance to TMZ are essential to devise treatment strategies to overcome resistance. Invasive GBM cells infiltrate healthy brain tissue, and are surrounded (and “protected”) by blood brain barrier (BBB) and blood brain tumour barrier (BBTB). BBB endothelial tight junctions form a physical barrier significantly reducing passive diffusion of drug molecules, therefore, efficacious doses of oral and *i.v.* chemotherapy fail to reach the brain^[8]^. Thus, tough challenges need to be surmounted before BBB penetration and drug transit to tumour sites are achieved^[9]^. Despite TMZ being able to cross the BBB relatively well via diffusion, it is estimated that < 1% of TMZ administered reaches the brain^[10]^. Active drug efflux pumps such as P-glycoprotein (P-gp), present on the BBB and tumour cells, and the short TMZ plasma half-life contribute to inefficient delivery to the brain. Further exacerbating efficacy is dose-limiting bone marrow toxicity associated with many anticancer chemotherapeutic agents.

According to the National Cancer Institute (NCI) https://www.cancer.gov/about-cancer/treatment/drugs/brain May 2019, FDA-approved drugs for GBM include: bevacizumab (Avastin); carmustine - and the carmustine implant, Gliadel wafers; lomustine; and TMZ [Table 1]. TMZ, the standard of care chemotherapy agent, lomustine and carmustine belong to the alkylating class of chemotherapy agents. Inherent and acquired resistance to this class pose further impediments to successful treatment. TMZ acts as a prodrug, stable at acidic pH, allowing oral administration^[11]^. At physiological pH TMZ undergoes ring opening (plasma t½ 1.8 h) to form monomethyl triazene 5-(3-methyltriazen-1-yl)-imidazole-4-carboxamide (MTIC). MTIC further reacts with water to liberate 5-aminoimidazole-4-carboxamide and a highly reactive methyldiazonium cation.

**Table 1:** Structures and growth inhibitory properties of TMZ and N-3 analogues

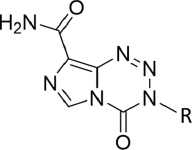		Cell line GI_50_ value (mean ± SD μmol/L)			
R	Compound	U373V	U373M	SNB19V	SNB19M
CH_3_	TMZ	51.9 ± 7	358.7 ± 26	35.7 ± 12	469.9 ± 42
CH_2_CO_2_CH_3_	Me-ester	32.1 ± 21	46.4 ± 16	47.5 ± 17	49 ± 14
CH_2_CO_2_CH_2_CH_3_	Et-ester				
CH_2_Si(CH_3_)_3_	TMS-methyl	45.6 ± 14	249.5 ± 35	28.4 ± 5.9	303.4 ± 34
CH_2_O(CH_2_)_2_Si(CH_3_)_3_	SEM	25.3 ± 3	24.2 ± 2.7	29.9 ± 5	27.5 ± 1.4
CH_2_OCH_3_	MOM	26.0 ± 5	33.1 ± 9.3	30.8 ± 9	32.8 ± 9
CH_2_SOCH_3_	Sulfoxide	8.2 ± 3.7	7.3 ± 1.8	28.9 ± 7.1	14.3 ± 6.8
CH_2_CHCH	Propargyl (N3P)	30.0 ± 2.3	26.6 ± 4	35.6 ± 14	36.1 ± 7

Means ± SDs ≥ 3 individual trials are shown; *n* = 4 per trial. TMZ: Temozolomide

**Figure 2:**
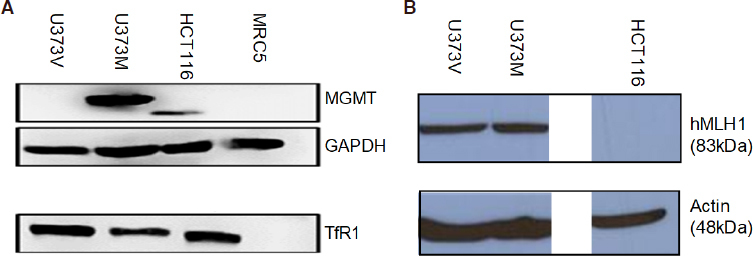
Expression of (A) MGMT and TfR1; (B) MMR protein hMLH1 in lysates prepared from U373V, U373M, HCT 116 cancer cell lines and MRC-5 fibroblasts. Proteins (50 μg) were separated by PAGE. Monoclonal antibodies were used to detect proteins of interest in addition to GAPDH and Actin to ensure equal loading and detection. Representative cropped blots are shown. Lysates were prepared on ≥ 3 separate occasions

The methyldiazonium cation preferentially methylates DNA at N7 positions of guanine in guanine rich regions (*N*7-MeG; comprising 70% of lesions), but also methylates N3 adenine (N3-MeA) and *O6* guanine residues^[12,13]^. *O*6-Methylguanine (*O*6-MeG; ~6% of all TMZ lesions) is the most cytotoxic lesion, but can be removed by *O*6-MeG-DNA methyltransferase (MGMT), a suicide direct repair enzyme. Epigenetic silencing of MGMT by gene promoter methylation compromises DNA repair and is associated with longer survival in patients receiving alkylating agent chemotherapy: Hegi *et al.*^[14]^ reported that in a cohort of 206 glioblastoma patients, MGMT was silenced in 45% GBM cases at diagnosis and confered longer survival in patients receiving TMZ. MGMT expression by GBM cells - either inherent or acquired following TMZ treatment confers resistance to TMZ *in vitro*^[15,16]^; and clinically^[17,18]^. A robust inverse relationship exists between MGMT expression and sensitivity to TMZ clinically and *in vitro* - as exemplified by U373M, a GBM cell line expressing MGMT [Figure 2]. GI_50_ values > 300 μmol/L reflect resistance to TMZ [Tables 1 and 2], whereas, its isogenic partner, U373V (expressing only the vector control) is ~6-fold more sensitive to TMZ. In the absence of MGMT, *O*6-MeG mispairs with thymine (T) - alerting DNA mismatch repair (MMR) and triggering DNA strand incision, removal of T, which is then replaced by T. During attempted repair S- and G2/M- cell cycle arrest can be observed ~72-120 h post TMZ treatment before this futile cycle leads eventually to cell death by apoptosis or autophagy^[19]^. DNA MMR deficiency, observed in 15%-20% sporadic colon carcinomas and Lynch syndrome, leads to tolerance of *O*6-MeG lesions, microsatellite instability^[20]^ and resistance to alkylating agents such as TMZ. HCT 116, a CRC carcinoma cell line lacking MMR protein hMLH1 [Figure 2] demonstrates resistance to TMZ [Table 2]. *In vitro*^[15]^, and clinically in GBM patients^[21,22]^, loss of MMR in response to TMZ treatment confers resistance to alkylating agents and insidiously, drives a hypermutator phenotype^[23,24]^.

**Table 2:** Growth inhibitory properties of TMZ, N3P and N3-sulfoxide against MMR-deficient CRC cell lines, TMZ-sensitive U87MG GBM, and GBM cell lines possessing acquired resistance to TMZ

	Mean GI_50_ value μmol/L				
	HCT 116 MLH1- MGMT+	DLD-1 MSH6- MGMT+	SNBVR MSH6- MGMT-	U373VR MMR+ MGMT+	U87MG MMR+ MGMT-low
TMZ	579.9	501.4	280.2	288.8	38.3
N3P	57.0	42.7	49.8	42.5	30.6
N3-sulfoxide	5.5	7.4	9.2	5.7	20.0

Means ≥ 3 individual trials are shown; *n* = 4 per trial. TMZ: Temozolomide; GBM: glioblastoma multiforme

Multiple molecular mechanisms contribute to drug-response or -resistance in gliomas, including expression of multi-drug resistance proteins (MRP)1, MRP3, MRP5 as well as P-gp and glutathione-*S*-transferase π^[25]^. Loss of heterozygosity on chromosome 10 is a frequent genetic abnormatlity observed in high grade gliomas (HGG), and an adverse prognostic factor in this disease^[26]^. Inherent resistance to apoptosis results from mutations of phosphate and tensin homologue on chromosome 10 (PTEN) tumour suppressor conferring constitutively active phosphatidylinositol-3-kinase (PI3K)/AKT/mammalian target of rapamycin and NF-κB signal transduction^[27]^. In paediatric glioma upregulation of HOX gene expression associated with PI3K- and mitogen-activated protein kinase - cascade activation has been detected^[28,29]^. Isocitrate dehydrogenase 1 and 2 (IDH 1/2) are key enzymes in cellular metabolism, epigenetic regulation, redox status and DNA repair^[30]^. In various tumour types, IDH mutations confer improved responses to radio- or chemotherapy. Common in grade 2 and 3 diffuse astrocytomas or oligodendromas, IDH mutations are prognostic for better clinical outcomes and longer patient survival. Poly [ADP-ribose] polymerase 1 (PARP-1)-associated DNA repair is compromised in IDH-mutant cells and TMZ treatment induced greater DNA damage in IDH-mutant glioma cells^[31]^. TMZ-generated *N*7-MeG, *N*3-MeA adducts are removed by base excision repair, facilitated by PARP-1, and a PARP-inhibitor/TMZ combination strategy has been shown to be an effective therapeutic approach for clinical management of advanced malignancies^[32,33]^. The cytotoxic *O*6-MeG DNA lesions wrought by TMZ are removed by MGMT and recognised by DNA MMR proteins. Clinically, therefore, MGMT expression and loss of MMR are most commonly responsible for failure of successful alkylating agent therapy.

## Novel temozlomide analogues

To address the unmet clinical need, we have synthesised TMZ analogues, initially modifying the N-3 position of the imidazotetrazine. The initial hypothesis under scrutiny being that an N3-alkylating moiety may be delivered to O6-guanine that is not removed by MGMT. Two isogenic GBM cell lines were employed in this study: SNB19M and U373M were stably transfected with MGMT; SNB19V and U373V were their vector control isogenic partners respectively. A number of analogues met this initial criterium [Table 1].

**Figure 3:**
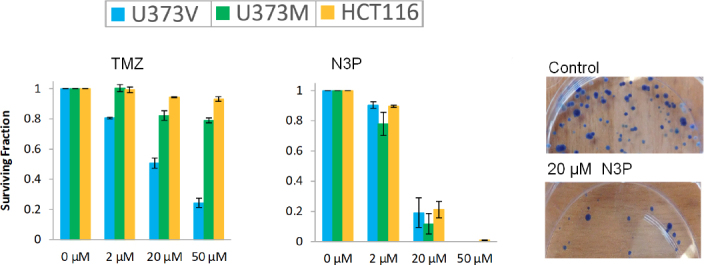
Clonogenic survival of U373V, U373M and HCT 116 cells following challenge (24 h) with temozolomide (TMZ) (A) and N3P (B); (C) representative HCT 116 colonies - control and following exposure of cells to 20 μmol/L N3P

Replacement of the N3-methyl group with methyl- or ethyl ester moieties gave compounds with modest activity irrespective of MGMT status^[34]^; however, *in vivo*, these analogues were devoid of activity, possibly because they are likely targets for hydrolysis by plasma esterases. Supporting this notion, the acetic acid derivative proved inactive against GBM cell lines. Interestingly, N3-trimethylsilyl (TMS)-methylimidazotetrazine showed an activity profile similar to TMZ (dependent on MGMT-absence); TMS-methyl lesions delivered to *O6*-guanine are good substrates for MGMT repair. In contrast, the N3-trimethylsilylethoxymethyl (SEM) analogue revealed surprising potency against MGMT+ cells (GI_50_ < 30 μmol/L), NMR studies indicated that the growth inhibitory activity may be a consequence of cytotoxic formaldehyde liberation within the cells^[35]^. Desirable potency irrespective of MGMT expression was achieved by substitution of N3-methyl with a range of polar alkyl groups such as methoxymethyl, sulfoxide and propargyl (N3P; Table 1). A second criterion, of critical clinical importance is delivery of an *O6*-guanine lesion that is not tolerated in the absence of DNA MMR. The N3-propargyl (N3P) and N3-sulfoxide imidazotetrazine analogues [Table 1] demonstrated activity in cell lines irrespective of MMR status; the growth of MMR-deficient CRC cell lines HCT 116 (lacking MLH1; Figure 2) and DLD 1 (MSH6-negative) cells was inhibited [Table 2]. Depicted in Figure 3 are clonogenic assays, performed to test whether GBM and CRC cells could survive a brief challenge with N3P imidazotetrazine analogue and proliferate to form progeny colonies. N3P conferred cytotoxic activity on cells irrespective of their MGMT or MMR status after only 24 h exposure. N3P at 50 μmol/L eradicated colony formation in U373V, U373M and HCT 116 cells. In contrast, TMZ (50 μmol/L) failed to significantly impact U373M and HCT 116 colony formation; only U373V clonogenic capacity was inhibited (~75%; Figure 3). Thus, clonogenic assays supported MTT results: cell lines with inherent resistance to TMZ (through repair or tolerance of *O*6-MeG mediated by MGMT presence or MMR deficiency respectively).

In addition, cell lines possessing acquired resistance to TMZ also demonstrated sensitivity to N3P and the N3-sulfoxide analogues [Table 2]. U373VR and SNB19VR were generated from vector control U373V and SNB19V GBM cell lines following long-term exposure to escalating TMZ concentrations; TMZ-resistance was acquired primarily through MGMT-upregulation and MMR-loss respectively^[15]^. Of particular note are GI_50_ values < 10 μmol/L following exposure of CRC and VR cell lines irrespective of MGMT or MMR status to N3-sulfoxide^[19]^. The activity (GI_50_ < 40 μmol/L) of N3P and N3-sulfoxide in the TMZ-sensitive (MGMT-low; MMR-proficient) U87MG GBM cell line is summarised. N3P and N3-sulfoxide analogues also showed promising activity in paediatric medulloblastoma cell lines expressing MGMT and P-gp-mediators of resistance^[36]^. Like TMZ, N3P and N3-sulfoxide were able to induce autophagic cell death in glioma cells resistant to apoptosis^[19]^.

In order to establish whether analogues possessed a similar mechanism of action to TMZ, it was necessary to synthesise and test *in vitro* activity of corresponding ring-opened triazene analogues. Faithful to their parent imidazotetrazines, MTIC was active only against vector control GBM cell lines; whereas, N3P triazene showed equi-potency (GI_50_ < 35 μmol/L) in GBM cell lines irrespective of MGMT status. However, the corresponding N3-sulfoxide triazene was highly unstable, and tests could not be performed.

**Figure 4:**
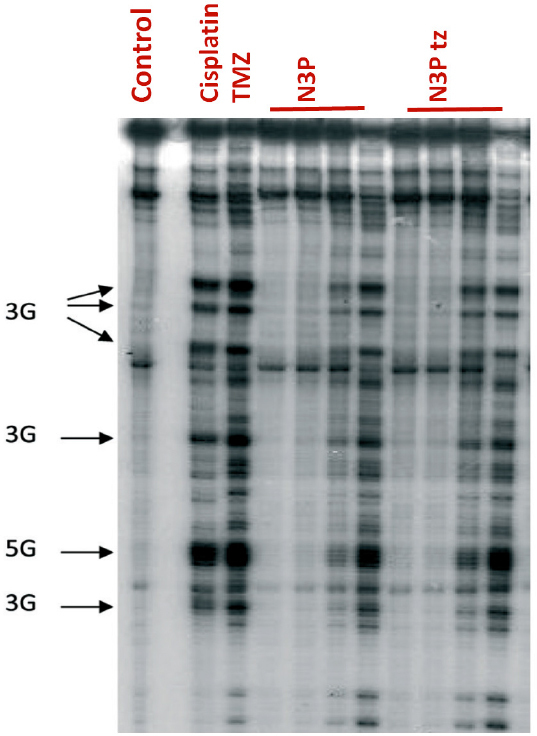
Autoradiogram of denaturing sequencing gel (Taq polymerase stop assay) showing alkylation sites induced by N3P and its ring-opened triazene (N3P tz). Control lane, unmodified BamHI fragment of pBR322 DNA; cisplatin (1 μm); Temozolomide (TMZ) (1000 μm); N3P (1 μm, 10 μm, 100 μm, 1000 μm); N3P tz (1 μm, 10 μm, 100 μm, 1000 μm). Arrows, indicate positions and sequence of binding sites on plasmid DNA. Drug-DNA incubations were for 2 h at 37 °C

In the *Thermus aquaticus* (TAQ) polymerase assays, N3P and its corresponding ring-opened triazene, like TMZ and MTIC alkylated plasmid pBR322 DNA at clusters of 3 and 5 contiguous guanine residues [Figure 4]. N3P does not cross link DNA and like TMZ, it is able to covalently modify N-7 sites of guanine, as evidenced in piperidine cleavage assays, and induce DNA double strand breaks^[37]^. These data provide evidence to support a mechanism of action similar to TMZ, i.e., a requirement for ring-opening prior to delivery of the propargyl lesion to purine sites. Therefore, the 3-propargyl substituent in imidazotetrazines confers potential for broader spectrum activity than TMZ.

However, preliminary *in vivo* efficacy studies carried out by colleagues at Schering-Plough corporation revealed disappointingly modest inhibition of TMZ-sensitive U87MG xenografts by N3P: whereas at 75 mg/kg TMZ inhibited tumour growth by 95%, 250 mg/kg N3P inhibited tumour growth by 54%. TMZ and N3P were administered *i.v.* on days 1-5 inclusive and tumour size analysed on day 11. Potential obstacles to efficacy include sub-optimum drug metabolism and pharmacokinetic properties (DMPK) and poor delivery of active agent to tumour tissue. Like TMZ, N3P is stable in acid (pH ≤ 5.5 t½ > 100 h), however, at physiological pH (7.4) a markedly shorter half-life has been recorded. In PBS, N3P had a t½ of 49 min compared to a t½ of 92 min for TMZ, potentially limiting delivery to the brain. Indeed, experiments demonstrated negligible N3P, or ring-opened N3P triazene penetrated the CNS.

To overcome this problem, two strategies have been adopted. The first strategy involved synthesis of TMZ analogues to test the hypothesis that modification of C-8 carboxamide will improve DMPK properties. Indeed, a recent publication presented evidence that the stability of the imidazotetrazine nucleus can be tuned by appropriate structural modifications at the C-8 position^[38]^. In our laboratory, a series of imidazo[5,1-d]-1,2,3,5-tetrazines bearing acyclic and cyclic modifications of the 8-carboxamide group, has been synthesised. The C-8 substituents included N-nitro- and -cyano-carboxamides, and a range of thiazole, benzoxazole, benzothiazole and benzimidazole rings^[39,40]^.

Of these, the C-8 imidazolyl analogues were of particular interest. Maintaining N3-methyl, but replacing C8-carboxamide with C8-imidazolyl (C8I) antitumour activity was observed in MGMT+ SNB19M and T989 GBM cells [Table 3] with 2-fold reduced potency compared to MGMT- SNB19V cells (IC_50_ ~33 μmol/L). Introduction of a methyl group on the imidazo group (C8-methylimidazole; C8MeI) resulted in enhanced potency (~2×) over C8I against GBM cell lines irrespective of MGMT status (IC_50_ values ~14 μmol/L and 32 μmol/L in SNB19V and SNB19M cells respectively). Therefore, MGMT, whilst conferring marked resistance to TMZ (C8 carboxamide), conferred only ~2-fold resistance to C8I and C8MeI analogues. Moreover, MMR-deficient HCT 116 cells responded to C8I and C8MeI N3-methyl TMZ analogues [Table 3]; consistently, C8MeI was twice as potent as C8I. Mechanistic studies revealed that similar to TMZ, C8I ring-opened liberating an intermediate that is more stable than MTIC. Furthermore, experiments using calf thymus DNA identified C8I-derived DNA adducts: *N*7-MeG, *N*3-MeA, *N*3-MeT, *N*3-MeC and, potentially critical for cytotoxicity, *O*6-MeG. Indeed, analogues arrested the cell cycle at G2/M phases irrespective of their MGMT or MMR status, subsequently, generating DNA double strand breaks and triggering apoptosis in carcinoma cell populations exposed to C8I and C8MeI^[39]^. It was concluded that these C8-imidazole analogues shared a mechanism of action similar to TMZ, but unlike TMZ were able in part to overcome both MGMT-mediated resistance and tolerance associated with MMR loss.

**Table 3:** Structures and growth inhibitory properties of TMZ and C-8 analogues

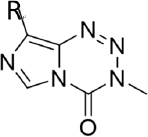		Cell line GI_50_ value (mean ± SD μmol/L)			
R	Compound	SNB19V	SNB19M	T98G	HCT 116
Carboxamide	TMZ	38.01 ± 16.35	508.84 ± 142	286.96 ± 19	592.88 ± 19.6
Imidazole	C8I	32.96 ± 10.8	65.92 ± 7.68	62.5 ± 6.9	44.25 ± 12.7
Me-Imidazole	C8MeI	14.34 ± 2.9	31.83 ± 8.6	33.09 ± 3.4	25.37 ± 2.2

Means ± SDs ≥ 3 individual trials are shown; *n* = 4 per trial^[39]^. TMZ: Temozolomide

## Apoferritin-encapsulated TMZ nanoparticles

The second strategy adopted to enhance imidazotetrazine efficacy and particularly BBB transit involves testing the hypothesis that by creation of imidazotetrazine nanoparticles, delivery to the brain can be prioritised. Exploitation of nanosized drug delivery systems (DDS) for targeted delivery to brain tumours is an approach adopted to encourage preferential drug accumulation within the brain tumour - enhancing efficacy, and minimising systemic toxicity^[41,42]^. For agents required to exert activity in the CNS, sufficient drug concentrations reaching the site in the clinical situation is a pre-requisite^[10]^. It has been estimated that < 1% drug administered reaches the brain. Indeed, following a single oral dose of TMZ (150 mg/m^2^), maximum plasma concentrations occur within 30-90 min. Ostermann *et al.*^[43]^ found that drug levels in the CSF achieved ~20% of those measured in systemic circulation; mean peak TMZ concentrations in the brain reach 0.6 ± 0.3 μg/mL^[44]^. An additional consequence of preferential drug delivery to the brain would be that reduced doses could be administered to effect therapeutic efficacy.

To enhance brain tumour accumulation and reduce systemic dose-related toxicity (thus improve efficacy and selectivity), we have employed apoferritin (AFt), as a nanosized DDS. Ferritins (Ft), ubiquitously expressed, are comprised of 24 protein subunits (heavy and light chains) in higher organisms; they self-assemble into hollow, spherical protein cages possessing outer and inner diameters of 12 nm and 8 nm respectively and 432 point symmetry^[45]^. Ft stores iron (≤ 4500 atoms per cage), protecting the cell from oxidative damage caused by the Fenton reaction, where in oxidising conditions Fe(II) can produce destructive reactive oxygen species. Upon removal of iron from the protein capsule, Ft becomes AFt^[46,47]^. AFt can be produced with high purity; it is biodegradable, biocompatible, non-immunogenic, of uniform size and architecture and robust stability, therefore considered an ideal DDS. The heavy chain (H) subunits of AFt bind to transferrin receptor 1 (TfR1)^[48]^ allowing H-Ft-mediated delivery of iron in times of high iron demand, such as neural development, rapid growth and cancer. Indeed, TfR1 is overexpressed in “iron-hungry” cancers^[49]^ (including gliomas), but not in mature oligodendrocytes^[50]^, and also heavily expressed by BBB endothelial cells^[51,52]^. TfR1 expression is limited to the endothelium of the brain (as opposed to peripheral endothelium)^[53]^, and the importance of H-Ft-mediated iron trafficking across the BBB^[54]^ and delivery to the brain^[55]^ has been demonstrated. We hypothesise that AFt-encapsulation of TMZ may enhance its delivery across the BBB, allowing evasion of membrane-mediated multi-drug resistance mechanisms such as P-gp expressed by BBB endothelial cells^[56]^; additionally, facilitating uptake of AFt-encapsulated cargo by cancer cells. Moreover, AFt-encapsulation of anticancer therapeutics such as imidazotetrazines may enhance drug solubility, bioavailability, protecting the encapsulated drug from elimination or biomolecular attack and protect the body from alkylating agent toxicity. Encouraged by successful encapsulation of clinical (gefitinib) and experimental (antitumour benzothiazoles) small molecules^[57,58]^, AFt-encapsulation of imidazotetrazines began.

**Figure 5:**
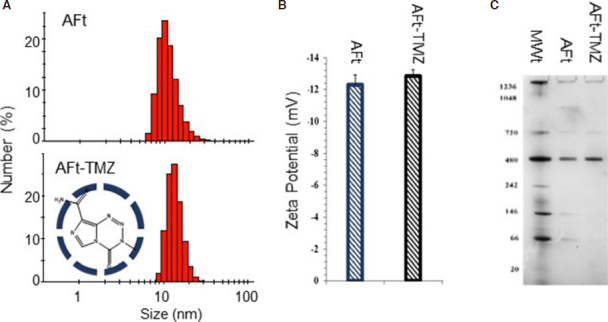
Characterization of AFt-TMZ. Hydrodynamic radius (A) and zeta potential (B) determination by dynamic light scattering (DLS) of AFt and AFt-TMZ; C: Native-PAGE molecular weight separation of AFt and AFt-TMZ using a 4%-16% gradient gel

The small size (MWt: 194) of TMZ allows its encapsulation via the nanoreactor route, where passive diffusion through the 0.3-0.4 nm channels between protein subunits is feasible. We have optimised the protocol reproducibly resulting in encapsulation of > 500 molecules of TMZ per AFt cage. The drug: AFt cage ratio, encapsulation efficiency (EE; > 80%) and drug loading (DL; > 18%) are summarised in Table 4. Development of this robust protocol that produces high concentrations of encapsulated TMZ has important implications potentially decreasing drug dose administered^[47]^. The integrity of the AFt cage post-encapsulation, and absence of drug-attachment to the AFt-exterior were confirmed by dynamic light scattering measurements. No detectable changes in hydrodynamic size and zeta potential were encountered [Figure 5], with average hydrodynamic diameter and zeta potential of 13.3 ± 0.83 nm and -12.7 ± 0.3 mV respectively. Native polyacrylamide gel electrophoresis (PAGE) of AFt-encapsulated TMZ exposed protein bands at MWt ~480 kDa and ~720 kDa - comparable to AFt alone, further corroborating the integrity of reformed AFt capsules. *In vitro* release studies of TMZ from AFt under physiologically-relevant conditions (pH 7.4 and pH 5.5 at 37 °C) revealed rapid release of TMZ during the initial 3 h. At pH 7.4, where imidazotetrazine ring-opening is more rapid, TMZ signal rapidly declined post 3 h. At pH 5.5 relaxation of the AFt cage is likely^[59]^, and maximal TMZ release (~85%) was evident by 8 h, TMZ could be detected at all time points monitored (≤ 24 h).

**Table 4:** Encapsulation of TMZ in 24-mer apoferritin protein cages

	TMZ-AFt ratio: No. molecules/cage	Encapsulation efficiency (EE%)	Drug loading (w/w%)
AFt-TMZ	527 ± 14	84.4 ± 5.2	18.7 ± 2.3

TMZ: Temozolomide; AFt: apoferritin

Having characterised AFt-encapsulation of TMZ, and confirmed drug liberation from the protein cages, *in vitro* antitumour tests were undertaken. MTT assays were initially employed to investigate antitumour activity of naked and AFt-encapsulated TMZ against isogenic GBM - U373V (vector control; MGMT-ve) and U373M (stably transfected with MGMT; MGMT+ve), paediatric SF188 high-grade glioma and DNA MMR-deficient CRC HCT 116 cancer cell lines as well as non-transformed MRC-5 fibroblasts. Work is underway on a larger panel of adult and paediatric glioma cell lines.

**Figure 6:**
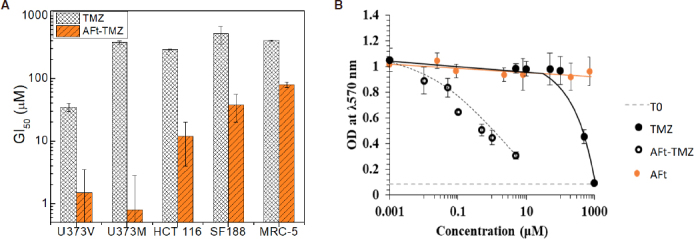
A: Bar chart illustrating growth inhibitory activity (GI50 values) of naked and apoferritin (AFt)-encapsulated temozolomide (TMZ) against U373V, U373M, SF188, HCT 116 cancer cell lines and MRC-5 fibroblasts. Means values ± SEM are shown if ≥ 3 independent trials; *n* = 4 per trial. B: Representative dose-response curves following exposure (6 d) of U373M cells to TMZ, AFt-TMZ or AFt alone

As illustrated in Figure 6A, AFt-encapsulation increases the potency of TMZ. Surprisingly, and of particular note is the marked enhanced potency of TMZ following AFt-encapsulation against MGMT+ U373M cells. Resistance to TMZ conferred by MGMT expression appears to have been overcome with GI_50_ values < 2 μmol/L, remarkably, > 250-fold enhanced potency compared to naked TMZ in this MGMT+ve GBM cell line. Figure 6B reveals representative dose-response profiles of TMZ, AFt-TMZ and AFt alone in U373M cells. As seen, AFt alone had no significant impact on cell growth or viability. It was also evident that, AFt-encapsulation of TMZ caused the MMR-deficient HCT 116 cell line to regain sensitivity to this alkylating agent (~25-fold reduction in GI_50_ value). Similarly encapsulation of TMZ in AFt cages restored sensitivity of SF188 paediatric glioma cells to TMZ. Resistance to TMZ is reported to be conferred by multiple mechanisms in SF 188 cells including PI3K signalling and MGMT expression^[28,29]^.

A degree of cancer cell-selectivity was encountered as AFt-TMZ possessed reduced activity against MRC5 fibroblasts compared to cancer cell lines [Figure 6A]. Interestingly, it can be seen that TfR1 expression, abundant in U373V, U373M, HCT 116 [Figure 2] and SF 188 (not shown) cancer cell lysates was undetectable in lysates prepared from MRC5 cells. Thus we may speculate that TfR1-mediated endocytosis of AFt-encapsulated cargo facilitates cellular internalisation of higher concentrations of TMZ. MGMT is a suicide repair enzyme and enhanced delivery of TMZ may overwhelm MGMT protein present - leading to the observed TMZ-sensitivity in the presence of MGMT. Indeed, this hypothesis is further supported by results obtained from MTT assays performed in MGMT+ paediatric brain tumour cell lines. DAOY (medulloblastoma) and BXD 1425 (ependymoma) cells were resistant to TMZ, but demonstrated sensitivity when challenged with AFt-encapsulated TMZ. In MMR-deficient HCT 116 cells, again, we speculate that TfR1 expression may facilitate increased AFt-TMZ cellular internalisation and that the subsequent incidence of *O*6-MeG-T DNA mis-pairs is too high to tolerate, triggering genome instability, cell cycle arrest and cell death^[60]^.

Clonogenic assays to examine the ability of single cells to survive 24 h exposure to TMZ or AFt-TMZ and produce progeny colonies have been performed; the clonal survival of U373M cells was impeded by 50 μmol/L TMZ only when administered as AFt-TMZ, naked TMZ had little impact on clonal survival. Cell cycle analyses have also demonstrated U373M arrest at S and G2/M phases following exposure to AFt-TMZ. These results support the hypothesis that enhanced cellular internalisation of TMZ overwhelms the suicide repair enzyme MGMT. Definitive assays are underway to rigorously test this theory, including: (1) measurement of DNA *O*6-MeG adducts in cells exposed to naked and AFt-TMZ; (2) phosphorylated (γ)-H2AX, a biomarker of DNA double strand breaks in cells exposed to imidazotetrazine analogues and AFt-formulations. Analyses of these results will inform design of subsequent *in vivo* studies to determine maximum tolerated dose, DMPK properties of novel TMZ analogues and AFt-encapsulated formulations, and their efficacy against subcutaneous and intra-cranial xenografts.

## Clinical implications

Because of the appalling prognoses associated with HGG such as GBM and DPIG, novel imidazotetrazine analogues or drug delivery formulations that can deliver an alkylating agent to *O6*-guanine that is neither removed by MGMT nor tolerated by MMR-deficient tumours are attractive prospects for future treatment. An initial study identified 55% GBM cases (in a study comprising 206 patients) where MGMT expression invalidated TMZ therapy^[14]^. In fact, multiple subsequent studies^[61]^ expose the hugely heterogenous nature of MGMT expression and activity (300-fold range). Undetectable MGMT activity was found in as few as 10% cases, and the frequency of MGMT-deficiency was 7-fold lower in tumours recurring after alkylating agent chemotherapy. Similarly predictive (more favourable) outcomes of MGMT gene silencing are seen in peadiatric brain tumours^[62,63]^. In peadiatric, as in adult gliomas, overexpression of MGMT is strongly associated with adverse outcome in children treated with alkylator-based chemotherapy independent of other clinical prognostic factors. MGMT is highly, and heterogeneously expressed in medulloblastoma, the most common malignant brain tumour in children: MGMT activity varied > 100-fold^[64,65]^. Clinically, loss of MMR proteins has arisen through Darwinian selection pressures imposed by TMZ treatment^[21,22]^, promoting a hypermutative phenotype^[23,24]^. Such genomic instability could lead to prevalence of secondary tumourigenesis, which, especially in children, should be avoided.

These stark facts and figures remind us of the unmet clinical need for novel agents and strategies to fight such intractable malignancies. To circumvent the BBB and evade systemic drug toxicities alternative drug-delivery strategies are being adopted. Intrathecal drug administration is a well-established route of delivery via the spinal canal or into the subarachnoid space to the cerebrospinal fluid to bypass the BBB. Simulating a more direct approach, intratumoural injection into flank tumours has been shown to enhance therapeutic effects^[66-68]^. One method under development, pioneered by Professor Steven Gill at The University of Bristol Medical School is convection-enhanced delivery (CED)^[69,70]^. CED uses direct intraparenchymal infusion to distribute drug specifically to the diseased brain area. Valproic acid and carboplatin have been administered by CED on compassionate grounds to children with lethal DIPG^[71]^ - a prelude to clinical trials. With collaborators at the University of Bristol, and the Children`s Brain Tumour Research Centre, University of Nottingham, we are investigating the feasibility of AFt-encapsulated formulations for CED.

## Conclusion

In conclusion, promising progress is being made to overcome inherent and acquired resistance to TMZ in intractable brain cancers. Two approaches have been attempted in our laboratories: (1) Analogues of TMZ have been synthesised that retain the same mode of action of TMZ but whose activity is not thwarted by MGMT presence or MMR deficiency [Tables 1, 2 and 3]. N3P and its corresponding triazene deliver alkylating lesions to guanine. C8I hydrolyses to liberate a stable intermediate that methylates *O6*-guanine resulting in GBM- and CRC cytotoxicity in the presence of MGMT and MMR-loss; (2) TMZ has been encapsulated within biocompatible AFt protein cages [Table 4] to enhance transit across the BBB and facilitate efficient delivery to the site of the tumour. Encouragingly, we encountered outstanding imidazotetrazine potency following AFt-encapsulation; surprisingly, AFt-TMZ overcame MGMT-mediated resistance, and demonstrated activity against MMR-deficient cancer cells. Our results to date inspire belief that with continued development of these strategies, the clinical prognoses for patients diagnosed with drug-resistant gliomas will be improved.

## Declarations

### Copyright

© The Author(s) 2019.
